# Mind and Milk

**Published:** 1874-01

**Authors:** 


### MIND AND MILK.

"MILK and water" people are of no account the world over ; they are of the smirky, namby-pamby kind, and the sooner they are all dead the better for them and everybody else. These agreeable stupidities, who assent to everything you do or say, who never did or could do anything but smile, away with them—they are of the "milk and water" flock ; they have no cream in them, nothing substantial, nothing nutritious or sustaining ; you might talk with them a whole year and never get a solitary idea.

It has been said for a long time that "It is the mind that makes the man." But although it cannot be exactly affirmed that milk makes the mind, yet it is true that the moral and mental character and physical condition of a man depend to a considerable extent on the milk he used while he was a baby. If the milk is rich in cream, and is made under favorable circumstances, both mind and body will the better for it. A young mother was thrown into a violent rage one day; in half an hour she became composed, and put her infant to the breast; in a few minutes it was in convulsions. Mrs. Henry Ward Beecher was a witness to the "transaction."

Farmers who supply the market with milk from their own cows know, from observation, that sometimes a cow will lose one half the percentage of cream in a day, all the surroundings being the same, except the condition of the cow's mind. Beat her, abuse her, worry her with dogs, do anything which keeps her in a state of unpleasant excitement, and the cream disappears apace.

A cow will give down her milk, and far richer milk, too, if you talk and laugh pleasantly, or pat her, or feed her, or sing to her, than if you curse, and swear, and kick at her all the time of milking.

Husband! will you make a manly note of this, and act accordingly towards the wife of your bosom in all her nursing days? that is, treat her with the highest possible respect-deference and courtesy, and thus let her have a consciousness of her womanhood all the time; meanwhile show her the utmost devotion and tenderness. Be a lover once more, say pleasant things, do pleasant things, arrange pleasant surroundings, provide pleasant surprises, and so manage that there shall be always around her an atmosphere of peace, and love, and elevation. That is the way to "grow" healthy, hearty, happy children. Try it once fairly.

